# The effect evaluation of Individual Placement and Support (IPS) for patients with substance use disorders: study protocol for a randomized controlled trial of IPS versus enhanced self-help

**DOI:** 10.1186/s13063-021-05673-z

**Published:** 2021-10-15

**Authors:** Eline Borger Rognli, Erlend Marius Aas, Robert E. Drake, John Marsden, Paul Anders, Gary R. Bond, June Ullevoldsæter Lystad, Silje Endresen Reme, Espen Ajo Arnevik

**Affiliations:** 1 Division of Mental Health and Addiction, Oslo University Hospital, P.O. Box 4956 Nydalen, 0424 Oslo, Norway; 2 Westat, Lebanon, New Hampshire USA; 3 IPS Employment Center, Laconia, New Hampshire USA; 4 Addictions Department, Institute of Psychiatry, King’s College London, London, UK; 5 Public Health England, London, UK; 6 Faculty of Social Sciences, University of Oslo, Oslo, Norway

**Keywords:** Substance use disorders, SUD, Individual Placement and Support, IPS, Employment support, Rehabilitation, Drugs, Dependence, Addiction, Treatment

## Abstract

**Background:**

Employment is associated with better outcomes of substance use treatment and protects against relapse after treatment completion. Unemployment rates are high for people with substance use disorders (SUD) who undergo treatment, with Norwegian estimates ranging from 81 to 91%. Evidence-based vocational models are lacking for patients in SUD treatment but exist for patients with psychosis in terms of Individual Placement and Support (IPS). The aim of the IPS for substance use disorders (IPS-SUD) trial is to investigate the effect of IPS in a SUD population.

**Methods/design:**

The IPS-SUD trial is a randomized controlled trial (RCT) comparing IPS to an enhanced control intervention. The study is a seven-site, two-arm, pragmatic, parallel-group, superiority RCT. Participants are randomly assigned (1:1) to receive either IPS plus treatment as usual (TAU) or to receive a self-help guide book and 12-h workshop plus 1-h individual vocational guidance plus TAU. Aiming to recruit 200 participants, we will be able to detect a 20% difference in the main outcome of employment with 90% power. We will make assessments at inclusion and at 6- and 12-month follow-ups and obtain outcome data on employment from national mandatory registries. The primary outcome will be at least 1 day of competitive employment during the 18-month follow-up period. Secondary employment outcomes will capture the pattern and extent of employment in terms of total time worked (days/hours), time to first employment, number of different jobs, duration of the longest employment, and sustained employment. Secondary non-employment outcomes will be substance use, mental distress, and quality of life measured by validated instruments at 6, 12, and 18 months follow-up assessments. To be eligible, participants must be between 18 and 65 years, currently unemployed and in treatment for SUD.

**Discussion:**

The IPS-SUD trial will provide evidence for the use of IPS in a SUD population. Findings from the study will have implications for service delivery.

**Trial registration:**

ClinicalTrials.gov NCT04289415. Registered on February 28, 2020

**Supplementary Information:**

The online version contains supplementary material available at 10.1186/s13063-021-05673-z.

## Background

Substance use disorders (SUDs) impose a significant burden in terms of individual suffering and societal cost [1]. Employment is associated with better outcomes of substance use treatment [2] and protects against relapse after treatment completion [3]. Reasons for this may be that having a job gives structure through the day, provides the person with a substance-free social network, and a stable economy. Having a job may also give a sense of dignity, belonging, and meaning—needs commonly expressed by patients with SUD but perhaps insufficiently met by the treatment system.

Evidence-based vocational methods are lacking for people with SUD [4, 5] but exist in other related areas. Individual Placement and Support (IPS) is an evidence-based psychosocial approach to help people with severe mental disorders obtain and keep competitive employment [6]. It is based on eight principles including a focus on competitive work and integration of vocational and clinical services in the same team, rapid job search, and ongoing in-work support. It emphasizes client job preferences and direct placement without prolonged pre-employment training [7]. Effective across a variety of settings and economic conditions, IPS is more than twice as likely to lead to competitive employment as traditional vocational rehabilitation methods [8]. After 20 years of accumulated evidence in favor of IPS for people with psychosis, researchers now call for IPS to be disseminated and tested on new target groups [9].

IPS may be effective for people with SUD, but this has not been sufficiently documented. Only one published randomized controlled trial has investigated the effect of IPS in a sample of people with SUD [10]. This American pilot study randomized 45 methadone patients to either IPS or a waiting list condition receiving treatment as usual (TAU). After 6 months, 50% of the participants in the IPS group had obtained employment versus 5% in the control condition. Another randomized controlled trial of the U.S. military veterans in the criminal justice system, in which 88% of the 84 study participants had a SUD, found significant results favoring IPS [11]. In addition, a meta-analysis demonstrated that IPS is effective for people with comorbid SUD in addition to severe mental disorders [12]. A large randomized controlled trial in the UK is currently investigating the effect of IPS in a SUD population [13]. Recruitment to the study has ended but results have not yet been published. A similar trial has been funded but not started in the USA (MDRC, Building Evidence on Employment Strategies (BEES) for Low-income Families, New York, 2020).

Norway has low unemployment rates, high job security, and a comprehensive welfare system. However, it also has the largest mental health-related unemployment gap of all OECD countries [14]. Mental and behavioral disorders, including SUDs, represent the most common reason for nonparticipation in the Norwegian labor market, accounting for 61% of all disability benefits among young adults in 2015 [15]. For people in SUD treatment, national unemployment rates are extremely high, ranging from 81 to 91% [16–19]. IPS has been proven effective in Norway for people with moderate and severe mental disorders [20, 21] and for young adults at risk of early work disability due to social- and health-related problems [22].

This article describes the protocol for the Individual Placement and Support for people with substance use disorders (IPS-SUD) trial.

## Methods

### Design

**Fig. 1:**
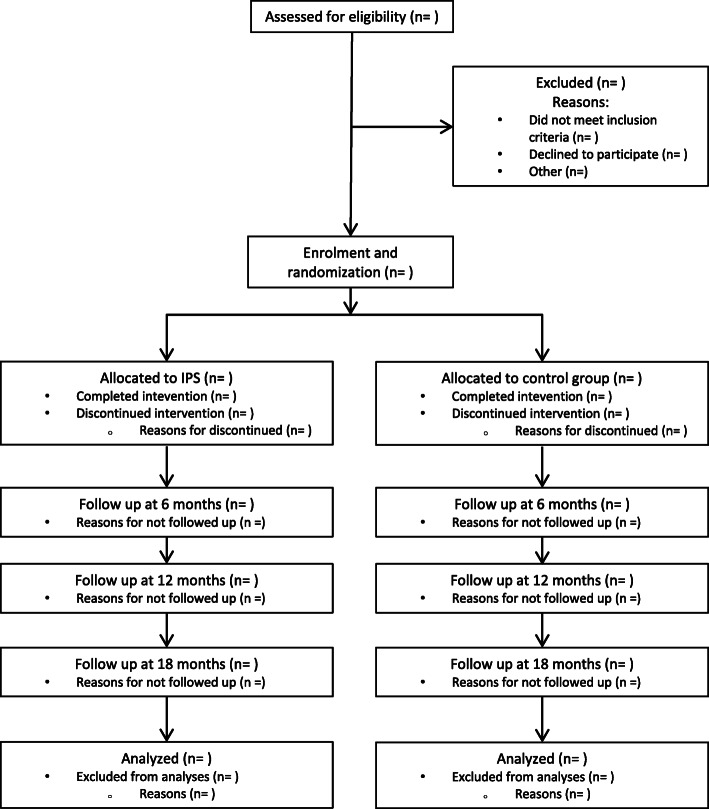
Consolidated standards of reporting trials (CONSORT) flow of participants

The IPS-SUD study is a seven-site, pragmatic, superiority randomized controlled trial with two arms. The aim of the study is to determine the effectiveness of IPS to aid people with SUD to obtain competitive employment. Participants will be randomly assigned with a 1:1 randomization ratio to either IPS plus TAU, or to a self-help guidebook and 12-h group-based workshop and 1-h individual consultation plus TAU. Assessments are made at baseline and at 6, 12, and 18 months of follow-up, and the primary outcome is obtained from a national registry at 18 months after inclusion in the trial (Fig. 1).

The study will be reported following the Consolidated Standards of Reporting Trials (CONSORT Guidelines) (http://www.consort-statement.org/) extension for non-medical trials [23]. This protocol has been written following the Standard Protocol Items: Recommendations for Interventional Trials (SPIRIT) Checklist for intervention trials [24] (see Additional file 1).

### Participants and study setting

We plan to recruit participants over 2 years, from March 1, 2020, to February 28, 2022. Patients will be recruited from five outpatient units and two inpatient units at the Department on Substance Use Disorder Treatment at Oslo University Hospital (OUS), Oslo, Norway. Two of the outpatient units are for patients undergoing opioid maintenance treatment (OMT) and serve the entire catchment area of OUS (twelve municipalities). Two outpatient units are local treatment centers serving the inhabitants of three municipalities each. The fifth outpatient unit serves the entire catchment area of OUS and offers some specialized services such as aid to users of anabolic-androgenic steroids. The two inpatient facilities offer treatment to patients with all types of SUD from all of OUS’s catchment areas; one for young patients (26 years and younger) and one for older patients (27 years and older). Due to higher patient turnover, it is expected that the majority of participants will come from the five outpatient units.

All treatment episodes at the study sites are by referrals. The referral must be sent by a health care professional (e.g., a general practitioner) and is evaluated by a team of experts who assess treatment needs and patient rights according to the law about access to specialized health care [25]. The treatment is mainly individual, but group-based interventions are also offered. Treatment usually consists of a combination of psychological, psychosocial, and pharmacological interventions. The duration of treatment at the outpatient units is around 1 year, but with large variations. The specialized health care system is responsible for the OMT patients as long as they receive their substitution treatment. The inpatient units offer 6 months of hospitalization, usually with outpatient follow-up.

### Inclusion and exclusion criteria

Inclusion criteria are (1) aged between 18 and 65 years, (2) in treatment for a SUD, (3) currently unemployed, (4) a wish to obtain competitive employment, and (5) able to communicate in Norwegian (not in need of interpreter). Exclusion criteria are (1) current treatment is coercive (under § 10.2, § 10.3 or § 10.4 in the Norwegian Act on Municipal health and care services), (2) treatment will be terminated within 1 year, and (3) previous (past 6 months) enrolment in the trial.

### Recruitment and randomization

All new patients entering treatment at the included sites will be assessed for eligibility by their clinician and asked if they are interested in competitive employment. If they respond yes, the clinician will provide written and verbal information about the research project. In addition to the new patients, patients who have been in treatment for a while but who will remain in treatment for a substantial period (for at least a year) will be assessed for eligibility and if eligible, invited to participate. This is particularly relevant for the OMT patients, as this in most cases is a lifelong treatment.

Patients interested in study participation will be referred to a research assistant, who will schedule an appointment with the potential participant. Here, further information will be given and the patient can ask any questions he or she has. Those who want to participate will sign an informed consent form, after which baseline assessment is conducted (Participant Information Sheet and Participant Consent Form are available from the corresponding author on request). The randomization will occur immediately after the baseline assessment, using a computer-generated randomization list which is an integrated part of the data capture system. Participants will be assigned by randomization to one of the two arms (ratio 1:1) using randomly varying block sizes and no stratification. The research assistant will immediately inform the participant about the result of the randomization. Patients assigned to the intervention group will be given the name of the employment specialist, who will contact the patient within a few days. Patients assigned to the control group will receive the self-help guidebook and an invitation with information and dates for the workshop. Workshops will be run regularly, and participants assigned to the control group will be invited to participate in the first upcoming workshop.

We acknowledge that there are factors that may slow down inclusion. Though there are few formal exclusion criteria for the trial, the clinicians may still apply their own assessments about whether their particular patient is ready and if he or she is likely to profit from study participation. Ongoing substance use or housing problems, though they are not exclusion criteria, may discourage the clinician to ask and inform the patient about the study. Also, the randomization and the possibility of being allocated to the control group may hold back some clinicians in approaching their patients, as they may want to protect their patients from a potential disappointment. Several steps will be applied to counter these challenges. Employment and eligibility for the project will be been made a routine topic in the clinical staff meetings where new patients are discussed. The employment specialists will be present at the clinical staff meeting and thereby provide a constant reminder of the study. Information about the study will be available as posters and leaflets in the waiting rooms. The project leader will have telephone contact with all the clinical unit leaders every second month or more often if needed, in order to identify potential challenges and needs for adjustment at an early stage. Brief updates on project status and inclusion will be sent to the unit leaders regularly. The leader group in the department will be informed about the project status every semester. The leader of the department is a member of the project steering committee.

### The IPS intervention

Participants will be referred to an IPS specialist, whose main goal is to help the patient, or job candidate, achieve employment of choice in the open job market. This will be done through intensive and personalized high-quality employment support, in this trial lasting up to 13 months. The duration of treatment in specialized healthcare for SUDs is usually around 1 year, and a previous study found no substantial difference in the effect of 13 months IPS versus time-unlimited IPS [26]. Also, in order to recruit a sufficient number of participants, we need a steady rate of terminations from IPS during the study enrolment period to make room on the IPS specialist’s caseloads for new study participants. The candidate and IPS specialist will together make a career profile based on the candidate’s strengths, experiences, and job preferences. The IPS specialist will aid in targeted job seeking and provide close follow-up support according to needs to both the employee and the employer once work is obtained. The IPS specialist will work systematically in networking and developing relations with local employers with the aim of identifying job opportunities for their candidates. The IPS team will be directly integrated with the health service, with the employment specialists located in the same office space as the health service, participating in the clients’ treatment teams and using a shared case management and documentation system. In order to give sufficient support, the IPS specialist’s client portfolio will have a capacity of maximum of 20 participants. In cases where new participants are hard to reach, the IPS-specialist will attempt repeatedly to establish contact over a period of 3 months, and if this is not successful, the IPS follow-up will stop.

A 25-item fidelity scale is used to evaluate the adherence of the service to the IPS model, with cut-offs for fair, good, and exemplary quality [27].

Fidelity reviews will be conducted by independent raters, and the first review will be done after the team has been operational for 6–12 months. The IPS team leader will have special responsibility for the methodological quality of the service.

When recruiting the IPS-team, we will seek people with experience in supported employment, preferably IPS, as well as experience in working with addiction and/or mental health. Before the first enrolment, the IPS team will receive standard training for IPS teams in terms of 3-day training in the IPS method, with additional 3-day training for the team leader. The training will be provided by the Norwegian Resource Centre for Community Mental Health (NAPHA) in collaboration with the Norwegian Welfare and Labour Administration (NAV) and the Norwegian Directorate of Health. In addition, the IPS team will have workshops covering basic knowledge in substance use and substance use treatment and motivational interviewing.

### The control group intervention

The control group will receive a self-help guidebook, with a supplemental workshop to aid the use of the guidebook. The guidebook will be given to the participant immediately after the randomization allocation. The workshop will take place in the facilities of the health service and consist of four 3-h sessions of didactic teaching and some practicing, and the number of participants will be between eight and twelve. Participants allocated to the control group will receive a reminder via text message the week before the workshop and the day before the first session. The participants will also be offered one session of individual consultation with the workshop facilitator. The goal is that the participants will be enabled to make use of the services of NAV, which are extensive but may feel inaccessible to this patient group.

The standard employment support provided by NAV may vary in its content, scope, and length, but consists of career counseling (individual or through workshops), help with job applications, and usually vocational training.

The schedule of enrolment, allocation interventions, and assessment is summarized in Table 1.

**Table 1:**
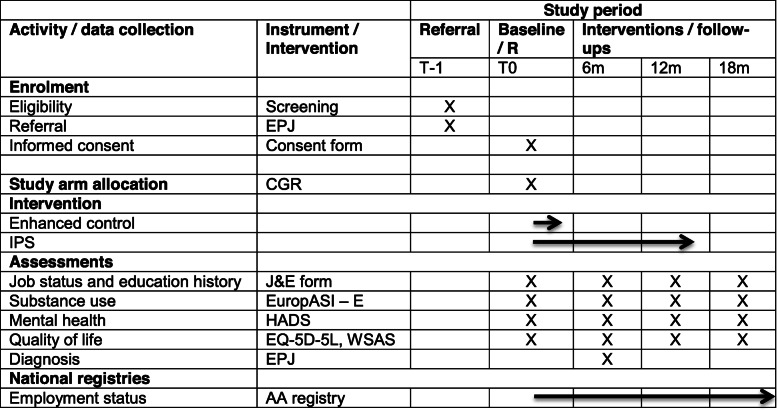
Schedule of enrolment, interventions, and assessments

*R* randomization, *EPJ* electronic patient journal system, *CGR* computer-generated randomization, *J&E form* job status and education history form (self developed), *EuropASI-E* E-section of European Addiction Severity Index, *HADS* Hospital Anxiety and Depression scale, *EQ-5D-5L* EuroQoL 5L - health-related quality of life, *WSAS* Work and Social Adjustment Scale, *AA registry* Norwegian national employer and employee register

### Sample size calculation

The so far only published randomized controlled trial of IPS for substance users had a 6-month follow-up and found large differences in employment between the two groups, 5% in the control group and 50% in the intervention group [10]. We find effects of this magnitude improbable in a Norwegian context with the extensive public employment service offered, and we base our assumptions on Norwegian data. A recent report on the status of substance users in the Oslo area states that 9% of the substance users in Oslo are employed [19]. In an IPS trial on moderate to severe mental disorders in Norway [21], employment was obtained for 37% in the intervention group. We use 37% as an estimate of the primary outcome for the intervention group. For the control group we assume 9% employment to begin with, but expect some effect of the workshop intervention, and thus increase our employment estimate for the control group to 15%. Setting an alpha level to 5%, with 90% power and group allocation ratio 1:1, we need 82 participants in each group to be able to detect a difference of 22% on the main outcome (odds ratio 3.33, 95% CI 1.68–6.59). By aiming for 100 participants in each group and a total sample size of 200, we will be able to detect a 20% difference on the main outcome with 90% power or a 17% difference with 80% power. A smaller difference in the main outcome is not considered clinically relevant, given the costs of the IPS intervention. We aim to recruit 100 participants in each group—a total of 200.

### Data collection

Baseline assessment and 6, 12, and 18 months of follow-up assessments will be done by one of the project’s two research assistants. Systematic methods for reminders for contacting patients for the follow-up assessments include telephone calls and text messages with a predefined content of the text messages as well as a predefined number of attempts to obtain contact with the participant. All baseline assessments will be done face-to-face, but in order to limit participant burden related to follow-up visits, assessment via telephone is an option if the participant does not wish to meet at the office building. As a financial reimbursement, participants receive a 250 NOK value gift card (approximately 25 US dollars) for follow-up interviews, handed out or sent as mail immediately after the follow-up interview. We will collect data using iPads with secure survey software, and data will be transferred directly to a secure server for the storage of research data at OUS.

The baseline assessment includes questions about background related to education, employment, welfare benefit reception, and housing condition. Some of the baseline questions recur at the follow-up assessments. At 6 months, we will also obtain diagnoses (primary and all secondary diagnoses) from the hospital records. We will use the following validated questionnaires to assess mental health status, substance use, and quality of life at baseline and follow-ups:

- Mental health status will be assessed by use of the Hospital Anxiety and Depression Scale (HADS) [28, 29], a 14-item questionnaire with a depression subscale and an anxiety subscale.
- Substance use history, past 6 months substance use, and past month substance use will be assessed with the E-section of EuropASI [30, 31].
- Quality of life will be assessed with the Work and Social Adjustment Scale (WSAS) [32, 33], a five-item questionnaire assessing the extent to which mental health problems have impaired functioning on five different domains during the past month; and with the EQ-5D-5L [34, 35], a five-item instrument which captures the health-related quality of life based on how the respondent feels on the day of completing the questionnaire. The EQ also contains a visual analog scale on which the respondent marks how he or she feels about his/her health on that particular day.

### Outcome measures

The primary outcome will be in accordance with most IPS-trials: at least 1 day of competitive employment during the 18-month follow-up period after inclusion in the trial. This operationalization, which includes very brief employment, turns out to be an excellent proxy for all dimensions of employment [36].

It provides a valid measure of the employment rate in randomized controlled studies. Secondary employment outcomes obtained at an 18-month follow-up will capture the pattern and extent of employment in terms of total time worked (days/hours), time to first employment, number of different jobs, duration of the longest employment, and sustained employment. Sustained employment will be defined as tenure in a single job for at least 13 weeks, to enable comparison to a UK trial also examining IPS for people with SUD [13].

We will use the State Register of Employers and Employees (the AA-registry) administered by Statistics Norway, as measures of employment. This is a mandatory registration system for employers, who provide information about their employees (starting date, full-time equivalent, hours per week for full position, and type of work) on a monthly basis. Reporting is conducted when an employee exceeds earnings of 1000 NOK per year (approximately 92 Euro or 105 USD) and is provided even if the employee did not receive payment that particular month. In addition, self-reported employment status is obtained at all data collection points, together with information about education, certificates, and other forms of preparatory steps the participant may take towards employment. Self-employment and work conducted “under-the-table” or in the black market will not be visible in the public register, but can be captured in the follow-up assessments. The outcome analyses on employment will be based on employment data from the AA registry, but sensitivity analyses using employment data from our follow-up assessments will also be conducted.

Secondary non-employment outcomes are substance use (past 6 months and past month), current mental health status, and current quality of life.

For dichotomous variables, we will compare the proportion between the two groups. For continuous variables, we will, depending on the variable distribution, consider presenting both mean and median, together with standard deviation, quartiles, and minimum-maximum values.

The consent form contains permission to link survey data with registries on the use of specialized and municipal health care and social welfare reception up to 10 years after inclusion, allowing for long-term, health-economic cost-benefit calculations.

### Study governance

The study is owned by the Section for Clinical Addiction Research at OUH. The Project Board is responsible for the general overview and progress of the trial and consists of site leaders and a representative from NAV Oslo as well as the trial leader and the leader of Section for Clinical Addiction Research. The Trial Management Group is responsible for the daily management and coordination of the trial. The Clinical Trials Unit at OUH is responsible for the management of data. A Scientific Advisory Group contributed with advice in the planning phase of the study and will be consulted through the trial when necessary. We have no plans to periodically review the accumulated data or conduct interim analysis while the trial is ongoing, and the trial does therefore not have a Data Monitoring Committee.

### Statistical analyses

The statistical analysis plan will be approved by the project board and pre-registered before data lock. Given the sensitive nature of the data, there are no current plans to make the dataset available for other researchers.

We will present baseline characteristics with regard to clinical, educational, and employment-related background and current status using descriptive statistics (mean, standard deviation, numbers, and percentages) for the sample as a whole. To investigate baseline balance, we will also present background characteristics and test differences according to the randomization group. The analyses of the primary outcome will follow the “intention to treat” principle and include all participants in the group to which they are allocated. Registry data will allow for nearly the entire enrolled sample in the follow-up analysis of the primary outcome.

Alpha levels for the primary and secondary outcomes will be set to 5% (with associated 95% confidence intervals).

For the primary outcome, data from the seven sites will be pooled, and the superiority effectiveness estimate for the IPS intervention (OR and CI) will be determined using a mixed-effects logistic regression model. The model will include a random intercept for each site to account for clustering. For secondary outcomes, continuous variables will be analyzed using linear mixed models. A time function will be included in the model to account for repeated measures.

There will be no missing data on the baseline measurements, as research assistants do the interviewing and answers on all questions are necessary to continue the electronic questionnaire. As data on employment will be obtained from a mandatory national register, we expect little or no missing data on the employment-related outcomes except for the under-the-table earnings. We do however expect attrition on the 6, 12, and 18 months surveys, affecting the secondary non-employment outcomes (substance use, mental distress, and quality of life). We will look at the pattern and proportion of missing data on these outcomes and handle the missing data according to recommendations for randomized controlled trials, preferably by means of multiple imputations [37, 38].

### Dissemination

The investigators will communicate results by submission of papers to international peer-reviewed journals. We will also make results available to healthcare professionals, stakeholders, and users through reports and oral presentations, and the results will be available on the project website.

### Confidentiality and ethical considerations

The project group will not receive any paper records. We will use Oslo University Hospital’s safe system for data capture and transference, and all data will be stored on a secure server used for research data at OUS. The list linking the project-generated ID number with the national personal identification number (PIN) will be stored on another safe OUS-server used for this purpose and will only be possible to access by the project leader and those the project leader gives access. Statistics Norway will conduct the linkage between our survey data and the registry-based employment data by means of the PIN, and when returning the dataset to the researchers, the PIN will be substituted by the project-generated ID number. The signed informed consent forms will be stored in a locked safe. The principal investigator and the research group at the study site will have access to the final trial dataset.

The trial has been evaluated and approved by the Norwegian Regional Ethical Committee (REC) (reference number 54204) and accepted by the Data Protection Officer at OUH. Decisions to amend the protocol will be made by the principal investigator together with the research group and the leader of the Project Board. Small amendments will be accumulated and sent to REC and substantive protocol amendments will be sent to REC immediately. The trial registry will be updated in the same manner. The project will adhere to the principles in the Helsinki declaration and the requirements issued by the General Data Protection Regulation. Participation is voluntary and based on informed consent. The participant information sheet following the informed consent explains that participants can withdraw from the study at any time without explanation and without the withdrawal affecting their substance use treatment. We consider the randomization procedure to be ethically acceptable since the effect of IPS is still largely unknown for this particular group, but chose for ethical and other reasons to provide a small, group-based intervention to the control group. This is a trial with minimal known risks and potential harms, and there are no predefined criteria for which the interventions will be modified or the trial discontinued. Adverse events will be registered in the hospital registration system for such events and reported to the trial board.

## Conclusion

The effectiveness evaluation of Individual Placement and Support for patients with substance use disorders will provide evidence for the use of IPS in this patient population. It will be the first randomized controlled trial to evaluate the effect of IPS for patients with SUD within a Norwegian context, and one of the first studies to do so internationally.

### Trial status

IPS-SUD was registered in the international trial register ClinicalTrials.gov on February 28, 2020 (identifier NCT04289415). This article refers to version 2.0 of the approved protocol (January 20, 2020). The first participant was enrolled in the study on March 4, 2020. The trial is ongoing and recruiting participants. The last day of participant inclusion will be February 28, 2022.

## Supplementary Information

**Additional file 1:.** SPIRIT 2013 Checklist: Recommended items to address in a clinical trial protocol and related documents*

## Data Availability

The project leader and the project group will have access to the final dataset. The dataset generated during the current study will not be publically available, as data sharing was not included in the protocol and consent form on which the ethical approval was based on.

## Abbreviations

IPS: Individual placement and support
NAV: Norwegian labour and welfare administration
OMT: Opioid maintenance treatment
OUH: Oslo university hospital
RCT: Randomized controlled trial
SUD: Substance use disorders
TAU: Treatment as usual
